# Prevalence and associated factors of hyperuricemia among urban adults aged 35–79 years in southwestern China: a community-based cross-sectional study

**DOI:** 10.1038/s41598-020-72780-3

**Published:** 2020-09-24

**Authors:** Xiao-Bo Huang, Wen-Qiang Zhang, Wei-Wei Tang, Ya Liu, Yuan Ning, Chuan Huang, Jian-Xiong Liu, Yan-Jing Yi, Rong-Hua Xu, Tzung-Dau Wang

**Affiliations:** 1 Department of Cardiology, The Second People’s Hospital of Chengdu, Chengdu, Sichuan China; 2 Department of Epidemiology and Health Statistics, West China School of Public Health and West China Fourth Hospital, Sichuan University, Chengdu, Sichuan China; 3 School of Health Policy and Management, Nanjing Medical University, Nanjing, Jiangsu Province China; 4 Center for Global Health, Nanjing Medical University, Nanjing, Jiangsu Province China; 5 Department of Endocrinology and Metabolism, Second People’s Hospital of Chengdu, Chengdu, Sichuan China; 6 Department of Endocrinology and Metabolism, Nanchong Central Hospital, Nanchong, Sichuan China; 7 Department of Geriatrics, Second People’s Hospital of Chengdu, Chengdu, Sichuan China; 8 Stroke Center, Second People’s Hospital of Chengdu, Chengdu, Sichuan China; 9 Division of Cardiology, Department of Internal Medicine, National Taiwan University Hospital, Taipei City, Taiwan

**Keywords:** Risk factors, Epidemiology, Metabolic syndrome

## Abstract

Hyperuricemia is prevalent throughout the world. However, a well-designed large-scale epidemiological investigation of hyperuricemia in southwestern China is lacking. A regional representative sample of 10,141 participants were included using multistage, stratified sampling in Chengdu and Chongqing from September 2013 to March 2014. Hyperuricemia was defined as the self-reported of the doctor's diagnosis of hyperuricemia, or serum uric acid > 420 μmol/L in men or serum uric acid > 360 μmol/L in women. The overall age- and sex-standardized prevalence of hyperuricemia among adults aged 35–79 years was 13.5%. Compared with women, the prevalence of hyperuricemia in men was higher (17.3% versus 10.0%). Hypertension, hyperlipidemia, overweight or obesity, central obesity were associated with an increased risk for hyperuricemia both in men and in women. Married men and women were not susceptible to hyperuricemia. Current cigarette smoking was an associated risk factor of hyperuricemia only in women. Hyperuricemia has become a major health problem among urban adults aged 35–79 years in southwestern China, and special attention should be paid to men. Comorbidities associated with hyperuricemia and causality worth further investigation.

## Introduction

Uric acid is a natural product generated from purine metabolism. Hyperuricemia can result from the overproduction or underexcretion of uric acid in human^1,2^. In the human evolutionary perspective, higher uric acid concentrations may have a survival advantage during the period of starvation in the past^3–5^. Although it is inconclusive that hyperuricemia is both a protective and causative factor in non-communicable diseases (e.g., cardiovascular disease^6,7^, neurodegenerative disease^8,9^), hyperuricemia is the cause of gout^1,3,10^. Elevated levels of serum uric acid (SUA) has been observed throughout the world, and hyperuricemia is prevalent both in developed and developing countries^11–15^.

Due to rapid industrialization, urbanization, and aging, the burden of non-communicable diseases is striking in China^16^. In 2009–2010, the prevalence of hyperuricemia among Chinese adults was 8.4%, approximately 92.9 million adults with hyperuricemia^14^. According to regional data in 2018 released from National Bureau of Statistics of China, more than 200 million people reside in southwestern China, approximately a seventh of the Chinese population, contributing to greater than 9520 billion CNY of GDPs. However, a well-designed large-scale epidemiological investigation of hyperuricemia in southwestern China is lacking, with one including 1458 subjects in Tibet Autonomous Region^17^, and the other including 1416 subjects in Ganzi Tibetan Autonomous Prefecture, Sichuan Province^18^. We aimed to conduct a large community-based cross-sectional study to assess the prevalence of hyperuricemia among urban adults aged 35–79 years in Chengdu and Chongqing to provide reliable and credible information for the development of a better prevention and control program for hyperuricemia in urban China.

## Results

### Participant characteristics

The demographic and clinical characteristics of the study participants are shown in Table 1. Of the 10,141 participants, 3447 were males and 6694 were females, and men had a higher mean age than women (*P* < 0.001). The majority (91.2%) of the study participants were married, but only 23.6% of the subjects had a high school education and above. Compared with women, men had higher education levels and higher personal monthly incomes (*P* < 0.001). Besides, men had a higher prevalence of drinking, smoking and regular exercise (*P* < 0.001). Men had higher WC, SBP, DBP, SUA (*P* < 0.001), while women had higher BMI, 2hPG, TC, HDL-C, LDL-C (*P* < 0.001). Moreover, women had higher TG than men (*P* = 0.006). There was no sex difference in FPG (*P* = 0.439).

**Table 1:** Demographic and clinical characteristics of the study participants.

	Overall (*n* = 10,141)	Male (*n* = 3447)	Female (*n* = 6694)	*P* value
Age (years)	55.0 ± 10.7	56.2 ± 10.9	54.3 ± 10.6	< 0.001
Married	9252 (91.2%)	3284 (95.3%)	5968 (89.2%)	< 0.001
High school education and above	2395 (23.6%)	1104 (32.0%)	1291 (19.3%)	< 0.001
Monthly income ≥ 2000 yuan	1912 (18.9%)	828 (24.0%)	1084 (16.2%)	< 0.001
Current cigarette smoking	2270 (22.4%)	2069 (60.0%)	201 (3.0%)	< 0.001
Alcohol drinking	203 (2.0%)	197 (5.7%)	6 (0.1%)	< 0.001
Regular physical exercise	423 (4.2%)	189 (5.5%)	234 (3.5%)	< 0.001
Hypertension	3754 (37.0%)	1338 (38.8%)	2416 (36.1%)	0.007
Diabetes mellitus	2093 (20.6%)	695 (20.2%)	1398 (20.9%)	0.395
Hyperlipidemia	3032 (29.9%)	1173 (34.0%)	1859 (27.8%)	< 0.001
Kidney disease	331 (3.3%)	48 (1.4%)	283 (4.2%)	< 0.001
BMI (kg/m^2^)	23.9 ± 3.5	23.6 ± 3.2	24.0 ± 3.6	< 0.001
WC (cm)	81.0 ± 10.4	82.6 ± 10.3	80.3 ± 10.4	< 0.001
SBP (mmHg)	130.8 ± 21.2	132.7 ± 20.0	129.8 ± 21.8	< 0.001
DBP (mmHg)	78.4 ± 11.2	80.4 ± 11.2	77.5 ± 11.2	< 0.001
FPG (mmol/L)	5.7 ± 1.8	5.7 ± 1.8	5.6 ± 1.7	0.439
2hPG (mmol/L)	7.9 ± 3.8	7.7 ± 3.8	8.0 ± 3.8	< 0.001
TG (mmol/L)	1.28 (0.91–1.86)	1.24 (0.88–1.87)	1.30 (0.92–1.85)	0.006
TC (mmol/L)	4.64 ± 0.93	4.50 ± 0.88	4.71 ± 0.94	< 0.001
LDL-C (mmol/L)	2.53 ± 0.75	2.47 ± 0.75	2.56 ± 0.75	< 0.001
HDL-C (mmol/L)	1.41 ± 0.34	1.34 ± 0.33	1.45 ± 0.34	< 0.001
SUA (μmol/L)	289.8 ± 81.0	340.8 ± 80.2	263.6 ± 67.8	< 0.001

Values are presented as mean ± standard deviation (SD), n (%); TG was reported as median (interquartile range). BMI, body mass index; WC, waist circumference; SBP, systolic blood pressure; DBP, diastolic blood pressure; FBG, fasting plasma glucose; 2hPG, 2-h plasma glucose; TG, triglyceride; TC, total cholesterol; LDL-C, low density lipoprotein cholesterol; HDL-C, high density lipoprotein cholesterol; SUA, serum uric acid.

### Serum uric acid level and hyperuricemia

**Figure 1:**
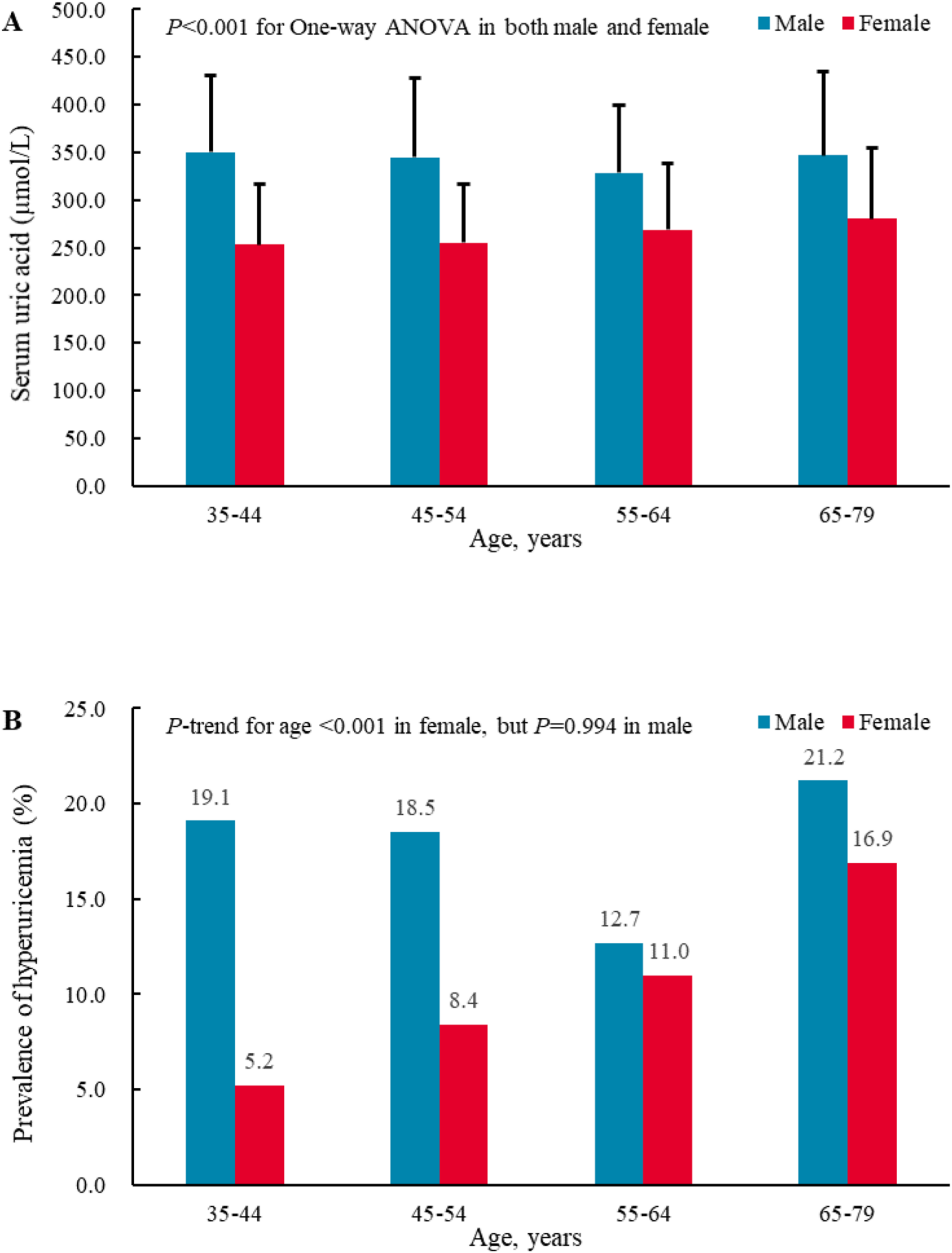
Age-specific serum uric acid level and prevalence of hyperuricemia among the adults aged ≥ 35 years in southwestern China.

As shown in Fig. 1A, the mean SUA level in men was 350.2 μmol/L (SD = 80.6) for aged 35–44 years, 344.9 μmol/L (SD = 82.8) for aged 45–54 years, 328.6 μmol/L (SD = 70.5) for aged 55–64 years, 346.7 μmol/L (SD = 88.0) for aged 65–79 years, respectively. In women, the mean SUA level was 253.4 μmol/L (SD = 64.0) for aged 35–44 years, 255.1 μmol/L (SD = 62.1) for aged 45–54 years, 269.1 μmol/L (SD = 69.0) for aged 55–64 years, 280.2 μmol/L (SD = 74.6) for aged 65–79 years, respectively. There was statistical significance in the SUA level between the age bands among men and women (*P* < 0.001). In addition, the crude overall prevalence of hyperuricemia among adults aged 35–79 years was 12.5%. The age- and sex-standardized prevalence of hyperuricemia was 13.5%. Compared with women, the prevalence of hyperuricemia in men was higher (17.3% versus 10.0%, *P* < 0.001). The prevalence of hyperuricemia increased with advancing age in women (*P* < 0.001, Fig. 1B). However, there was no increasing trend of hyperuricemia prevalence between the age bands in men (*P* = 0.994).

### Comorbidities of hyperuricemia

Among persons affected with hyperuricemia (Table 2), 55.3%, 27.4, 44.1%, 61.1%, 44.3% had hypertension, diabetes mellitus, hyperlipidemia, overweight/obesity, and central obesity, respectively. Findings stratified by sex indicated 56.0%, 21.7%, 41.8%, 58.7%, 36.6% of men with hyperuricemia had hypertension, diabetes mellitus, hyperlipidemia, overweight/obesity, and central obesity, respectively. 54.6%, 32.5%, 46.7%, 63.6%, 51.2% of women with hyperuricemia had hypertension, diabetes mellitus, hyperlipidemia, overweight/obesity, and central obesity, respectively.

**Table 2:** Comorbidities of hyperuricemia among the study participants by sex.

	Overall (*n* = 1268)	Male (*n* = 598)	Female (*n* = 670)
Hypertension	701 (55.3%)	335 (56.0%)	366 (54.6%)
Diabetes mellitus	348 (27.4%)	130 (21.7%)	218 (32.5%)
Hyperlipidemia	559 (44.1%)	280 (41.8%)	279 (46.7%)
Overweight/obesity	775 (61.1%)	351 (58.7%)	424 (63.6%)
Central obesity	562 (44.3%)	219 (36.6%)	343 (51.2%)

Values are presented as n (%).

### Associated factors of hyperuricemia

In the univariable logistic regression model analysis (Table 3), age, marital status, comorbidities of hypertension, hyperlipidemia, kidney disease, overweight or obesity, central obesity, were associated with the risk of hyperuricemia. In women, age, marital status, education, smoking, comorbidities of hypertension, diabetes mellitus, hyperlipidemia, overweight or obesity, central obesity, menopause, were associated with the risk of hyperuricemia. The multivariable logistic regression model showed that hypertension, hyperlipidemia, overweight or obesity, central obesity were positively associated with hyperuricemia, and being married was negatively associated with hyperuricemia in men. Compared with aged 35–44 years, people aged 55–64 years had lower risks of hyperuricemia in men. In women, advanced age, current cigarette smoking, hypertension, diabetes mellitus, hyperlipidemia, overweight or obesity, central obesity, menopause, were positively associated with higher risks of hyperuricemia. However, being married had lower risks of hyperuricemia than being divorced, widowed, or single.

**Table 3:** Odds Ratios for hyperuricemia among the study participants by sex.

	Male		Female	
	Model 1	Model 2	Model 1	Model 2
**Age groups**				
35–44 years	1.00 (reference)	1.00 (reference)	1.00 (reference)	1.00 (reference)
45–54 years	0.96 (0.74–1.26)	0.92 (0.70–1.22)	1.66 (1.25–2.19)^†^	1.45 (1.09–1.94)^†^
55–64 years	0.62 (0.48–0.80)^†^	0.52 (0.40–0.68)^†^	2.23 (1.72–2.90)^†^	1.40 (1.04–1.88)^†^
65–79 years	1.14 (0.87–1.46)	0.85 (0.64–1.11)	3.68 (2.81–4.83)^†^	1.76 (1.29–2.40)^†^
Married	0.57 (0.40–0.81)^†^	0.52 (0.36–0.76)^†^	0.51 (0.41–0.64)^†^	0.61 (0.49–0.77)^†^
High school education and above	1.18 (0.98–1.42)	–	0.72 (0.59–0.90)^†^	–
Monthly income ≥ 2000 yuan	0.97 (0.79–1.19)	–	0.96 (0.77–1.19)	–
Current cigarette smoking	1.05 (0.88–1.26)	–	1.81 (1.23–2.65)^†^	1.54 (1.02–2.33)^†^
Alcohol drinking*	1.36 (0.96–1.92)	–	–	–
Regular physical exercise	0.71 (0.46–1.10)	–	0.88 (0.56–1.39)	–
Hypertension	2.34 (1.96–2.80)^†^	2.17 (1.78–2.64)^†^	2.33 (1.99–2.74)^†^	1.49 (1.25–1.79)^†^
Diabetes mellitus	1.12 (0.91–1.39)	–	1.98 (1.66–2.36)^†^	1.23 (1.02–1.49)^†^
Hyperlipidemia	2.43 (2.02–2.91)^†^	2.01 (1.66–2.43)^†^	2.24 (1.90–2.64)^†^	1.76 (1.48–2.09)^†^
Kidney disease	1.98 (1.06–3.72)v	–	0.99 (0.66–1.47)	–
Overweight/obesity	2.13 (1.78–2.55)^†^	1.51 (1.22–1.87)^†^	2.12 (1.80–2.50)v	1.49 (1.22–1.81)^†^
Central obesity	2.08 (1.72–2.51)^†^	1.33 (1.06–1.67)^†^	2.23 (1.90–2.62)^†^	1.33 (1.09–1.61)^†^
**Menopause**				
Premenopausal	–	–	1.00 (reference)	1.00 (reference)
Postmenopausal	–	–	2.70 (2.14–3.40)^†^	1.56 (1.19–2.05)^†^
Missing 210	–	–	–	–

Values are presented as odds ratios (95% confidence interval). Model 1 was a univariable logistic regression model. Model 2 was a multivariable logistic regression model, using a forward-stepwise selection method (Likelihood Ratio, LR) to specify how independent variables are entered into the model.

*There were 6 persons defined as alcohol drinking in women, so it terminated.

^†^*P* < 0.05.

## Discussion

This large-scale population-based study from southwestern China was designed to investigate hyperuricemia and associated risk factors in urban adults aged 35–79 years in Chengdu and Chongqing from September 2013 to March 2014. Overall, the age- and sex-standardized prevalence of hyperuricemia was 13.5%. We showed differences in hyperuricemia prevalence between men (17.3%) and women (10.0%). Associated risk factors for hyperuricemia in men included hypertension, hyperlipidemia, overweight or obesity, central obesity. Advanced age, current cigarette smoking, hypertension, diabetes mellitus, hyperlipidemia, overweight or obesity, central obesity, menopause were identified as associated risk factors for hyperuricemia in women. Furthermore, being married was an associated protective factor both in men and women, although to varying degrees.

In this study, our estimated prevalence of hyperuricemia among adults aged 35–79 years is higher than the national estimated prevalence of hyperuricemia. Data from the China National Survey of Chronic Kidney Disease in 2009–2010 showed the prevalence of hyperuricemia among Chinese adult aged 18 years and above was 8.4%^14^. A larger national investigation from the China Health and Retirement Longitudinal Study in 2011 indicated the prevalence of hyperuricemia among adults aged 45 years and above was 6.4%^15^. Differences in the prevalence of hyperuricemia are in part due to the different socioeconomic context, age composition of subjects enrolled. As previous studies shown, the prevalence of hyperuricemia among Chinese adults is higher in economically developed areas and urban areas^14,15^. Thus, it is not surprising that the result in this study is much higher than the estimated prevalence using national investigations included less-developed areas and rural areas^14,15^.

The serum uric acid concentrations and the prevalence of hyperuricemia were higher in men than in women, verified in different studies^12–15^. The potential biological mechanism underlying the differences between the sexes might be the uricosuric effects of estrogen in premenopausal women^3,10,19^. Following the menopause, serum uric acid concentrations will increase in women^3,10^. As indicated in this study and previous studies, menopause is an associated risk factor of hyperuricemia in women, independently of age and other covariates^19,20^. Furthermore, the age-associated increase in the prevalence of hyperuricemia in women might be partly explained by menopause, and other age-related factors need more evidence to be confirmed^20^. However, the effect of advanced age on hyperuricemia in men was not observed in this study, which is not consistent from other studies^15,21–23^. The lowest prevalence of hyperuricemia was observed in men aged 55–64 years, identical with a previous study^24^, which might be associated with the phase of retirement and more attention on health in Chinese men ^24^. More prospective cohort studies should be conducted to confirm the risk of hyperuricemia caused by advanced age in men.

Some epidemiological studies have shown that cigarette smoking was associated with lower levels of serum uric acid, as a result of oxidative stress induced by the long-term exposure to cigarette smoking^25–27^. In this study, current cigarette smoking was an associated risk factor of hyperuricemia in women rather than men, although to varying degrees, consistent with the result from the China Health and Retirement Longitudinal Study in 2011^15^. The disparity might be elucidated by fewer pack-years in women^15,28^. Moreover, married men and women were not susceptible to hyperuricemia. The protective effect of marriage on risk of hyperuricemia may result from spousal interaction on health monitoring (e.g., health behavior, dietary pattern)^29,30^.

Similar to previous observational epidemiological studies, hypertension, hyperlipidemia, overweight or obesity, central obesity were associated with an increased risk for hyperuricemia^14,15,22,23,31,32^. Besides, diabetes mellitus was found to be associated with hyperuricemia only in women. In contrast, some studies have shown that higher serum uric acid was an associated risk factor for hypertension, diabetes mellitus, and obesity^33–37^. However, Mendelian randomization studies do not support the causality between serum uric acid and hypertension, diabetes mellitus^38–41^. Thus, further studies are still needed to confirm whether there is the bi-direction causality between hyperuricemia and cardiovascular diseases risk factors (e.g., hypertension, diabetes mellitus).

Because this study was cross sectional, we might suffer from many potential biases or reverse causation between associated risk factors and hyperuricemia. Some significant information was not available, such as dietary intake information and family history of hyperuricemia. Kidney disease was based on self-reported history, rather than estimated glomerular filtration rate, which might cause a false-negative result. About 24% of participants with missing values were excluded, which may cause potential selection bias. To minimize this bias, we computed the age- and sex-standardized prevalence using the 2010 census data of China. Finally, participants were enrolled from urban adults in Chengdu and Chongqing, which is likely to overestimate the prevalence of hyperuricemia among adults in southwestern China.

## Conclusions

Hyperuricemia has become a major health problem among urban adults aged 35–79 years in southwestern China, and special attention should be paid to men. Comorbidities associated with hyperuricemia and causality worth further investigation.

## Methods

### Participants

The study protocol has been published, approved by the ethics committee of the Second People’s Hospital of Chengdu (NO 2013015), the methods in the study were in accordance with relevant guidelines, and a written informed consent was obtained from all participants^42^. Using three-stage (district-subdistrict-community) sampling procedures, five representative urban communities were randomly selected from five districts, including Jinjiang, Qingyang, Longquan district in Chengdu, and Yubei, Jiangbei district in Chongqing. The inclusion criteria for this study were residents aged 35–79 years who had lived in the community for more than five years. The exclusion criteria were people with histories of secondary hypertension, mental illness, malignant tumors, renal failure requiring dialysis, or who refused to participate in the inquiry. In total, 13,378 participants were enrolled using multistage, stratified sampling, from September 2013 to March 2014. Data collection was completed by more than 30 trained investigators. A structured questionnaire and anthropometric measurements were conducted, including demographic characteristics (i.e. sex, age, education, marital status), health-related lifestyles (i.e. current cigarette smoking, alcohol drinking, regular physical exercise), chronic disorders (i.e. hypertension, diabetes, dyslipidemia, hyperuricemia), blood biomarkers (i.e. serum uric acid, fasting plasma glucose). We excluded 3237 participants, who had missing demographic characteristics (i.e. income, marital status), anthropometric measurement parameters (i.e. blood pressure, height), blood biomarkers (i.e. serum uric acid, fast blood glucose). Hence, we included 10,141 participants in the study.

### Diagnostic standards

According to Dietary guide for hyperuricemia and gout patients (Chinese standard, WS/T 560-2017), hyperuricemia was defined as serum uric acid > 420 μmol/L in men or serum uric acid > 360 μmol/L in women. The self-reported of the doctor's diagnosis of hyperuricemia was also diagnosed with hyperuricemia. Current cigarette smoking was defined as having smoked more than 100 cigarettes in one’s lifetime. Alcohol drinking was defined as consumption of more than 30 g of alcohol per week for more than 1 year. Regular physical exercise was defined as participation in more than 30 min of moderate or vigorous activity per day for more than 3 days per week^43^. Hypertension was defined as the self-reported history of hypertension or systolic blood pressure ≥ 140 mmHg and (or) diastolic blood pressure ≥ 90 mmHg. Diabetes was defined as the self-reported history of diabetes or fasting blood glucose ≥ 7.0 mmol/L and (or) OGTT 2-h post-load glucose ≥ 11.1 mmol/L. Based on the Criteria of weight for adults (Chinese standard, WS/T 428-2013), general obesity was determined by the BMI, categorized using the Chinese specific cutoff values as underweight (< 18.5), normal (18.5–23.9), overweight (24.0–27.9) and obesity (≥ 28.0). Central obesity was defined as WC ≥ 90 cm for men and ≥ 85 cm for women. Dyslipidemia was defined as total cholesterol ≥ 6.2 mmol/L, and/or LDL cholesterol ≥ 4.1 mmol/L, and/or HDL cholesterol < 1.0 mmol/L, and/or TG ≥ 2.3 mmol/L, and/or the self-reported history of dyslipidemia, based on the Chinese guidelines for the management of dyslipidemia in adults^44^. History of kidney disease was defined as the self-reported of the doctor's diagnosis of kidney disease. In women, menopause was defined as her menstrual periods had stopped at least 1 year.

### Statistical analysis

Categorical data were presented as absolute numbers with the percentage, and Pearson’s *Chi*-Square test was used to detect the difference between the sexes. Except for triglyceride (TG), continuous data were presented as means with the standard deviation (SD), and Student’s *t* test was used to detect the difference between the sexes. Triglyceride was presented as medians with the interquartile range because of its skewed distribution, and the Wilcoxon rank sum test was used to detect the difference between the sexes. Besides, one-way analysis of variance (ANOVA) was used to detect the difference in serum uric acid level between the age bands, and the Cochran-Armitage test was used to test the trend in hyperuricemia prevalence between the age bands. A univariable logistic regression model and a multivariable logistic regression model were used to estimate the odds ratios and 95% confidence interval to explore the associated risk factors of hyperuricemia. For menopause in women, there were 210 missing values, so we created a dump variable for missing values. All statistical analyses were performed using Statistical Product and Service Solutions (SPSS, version 23.0).
